# Harnessing symbiotic plant–fungus interactions to unleash hidden forces from extreme plant ecosystems

**DOI:** 10.1093/jxb/eraa040

**Published:** 2020-01-24

**Authors:** Marta-Marina Pérez-Alonso, Carmen Guerrero-Galán, Sandra S Scholz, Takatoshi Kiba, Hitoshi Sakakibara, Jutta Ludwig-Müller, Anne Krapp, Ralf Oelmüller, Jesús Vicente-Carbajosa, Stephan Pollmann

**Affiliations:** 1 Centro de Biotecnología y Genómica de Plantas, Universidad Politécnica de Madrid (UPM)–Instituto Nacional de Investigación y Tecnología Agraria y Alimentación (INIA), Campus de Montegancedo, Pozuelo de Alarcón (Madrid), Spain; 2 Matthias Schleiden Institute of Genetics, Bioinformatics and Molecular Botany, Department of Plant Physiology, Friedrich-Schiller-University Jena, Jena, Germany; 3 RIKEN Center for Sustainable Resource Science, Suehiro, Tsurumi, Yokohama, Japan; 4 Department of Applied Biosciences, Graduate School of Bioagricultural Sciences, Nagoya University, Nagoya, Japan; 5 Institute of Botany, Technische Universität Dresden, Dresden, Germany; 6 Institut Jean-Pierre Bourgin, INRA, AgroParisTech, CNRS, Université Paris-Saclay, Versailles, France; 7 Departamento de Biotecnología-Biología Vegetal, Escuela Técnica Superior de Ingeniería Agronómica, Alimentaria y de Biosistemas, Universidad Politécnica de Madrid (UPM), Madrid, Spain; 8 University of Edinburgh, UK

**Keywords:** Abiotic stress, crosstalk, endosymbiosis, plant performance, *Serendipita indica*

## Abstract

Global climate change is arguably one of the biggest threats of modern times and has already led to a wide range of impacts on the environment, economy, and society. Owing to past emissions and climate system inertia, global climate change is predicted to continue for decades even if anthropogenic greenhouse gas emissions were to stop immediately. In many regions, such as central Europe and the Mediterranean region, the temperature is likely to rise by 2–5 °C and annual precipitation is predicted to decrease. Expected heat and drought periods followed by floods, and unpredictable growing seasons, are predicted to have detrimental effects on agricultural production systems, causing immense economic losses and food supply problems. To mitigate the risks of climate change, agricultural innovations counteracting these effects need to be embraced and accelerated. To achieve maximum improvement, the required agricultural innovations should not focus only on crops but rather pursue a holistic approach including the entire ecosystem. Over millions of years, plants have evolved in close association with other organisms, particularly soil microbes that have shaped their evolution and contemporary ecology. Many studies have already highlighted beneficial interactions among plants and the communities of microorganisms with which they coexist. Questions arising from these discoveries are whether it will be possible to decipher a common molecular pattern and the underlying biochemical framework of interspecies communication, and whether such knowledge can be used to improve agricultural performance under environmental stress conditions. In this review, we summarize the current knowledge of plant interactions with fungal endosymbionts found in extreme ecosystems. Special attention will be paid to the interaction of plants with the symbiotic root-colonizing endophytic fungus *Serendipita indica*, which has been developed as a model system for beneficial plant–fungus interactions.

## Teaser

*Serendipita indica* can colonize a broad range of hosts, conferring improved stress tolerance and performance under adverse conditions. The underlying molecular mechanisms have the potential to improve agricultural practices in a climate change scenario.

## Introduction

Agriculture is the principal means of livelihood in many regions of the world. However, current agricultural practice is not sustainable and is known to cause serious ecological damage, such as soil erosion, eutrophication of water bodies, increasing salinity of soils, and desertification, which in the long run translates into substantially reduced agricultural productivity owing to further loss of arable land. Global climate change represents an additional hazard that aggravates the negative effects of current agricultural practices and causes further dramatic losses in agricultural production.

Given the increasing demand for agricultural products resulting from the growing world population, which is predicted to reach 9.5 billion people by 2050 (ISAAA, 2017), the combination of non-sustainable agricultural practices and climate change effects will not only entail economic losses for the agricultural industry but put the general food supply for humankind in considerable jeopardy. With respect to current estimates, 800–925 million people will be undernourished in 2020; ‘hidden hunger’ due to a lack of vitamins and minerals is estimated to be the most common form of malnutrition, affecting approximately 2 billion people (FAO *et al*., 2015; Fedoroff, 2015). Hence, immediate measures must be taken to limit the damage already caused to the environment and to secure food availability in the future. Advanced agricultural biotechnology methods, including the so-called ‘new breeding techniques’ consisting of directed genome editing (i.e. CRISPR/Cas), are expected to be able to alleviate the effects of climate change and to ensure a more sustainable agriculture (Aglawe *et al.*, 2018). These approaches include, for example, the generation of more drought- and salt-tolerant crops, or plants with improved nitrogen use efficiency or pathogen resistance, by cloning genes from plants found in high-stress environments into stress-sensitive but highly productive species. It is anticipated that these actions will reduce the application of pesticides and mineral fertilizers and limit the need for irrigation (Gartland and Gartland, 2018). According to our estimations, such exclusively plant-focused strategies might be insufficient to solve the issue of food security entirely. Thus, in this review we would like to draw attention to the symbiosis of plants with commensal microorganisms, an often-underestimated factor that can substantially affect plant performance under unfavorable growth conditions.

**Fig. 1.**
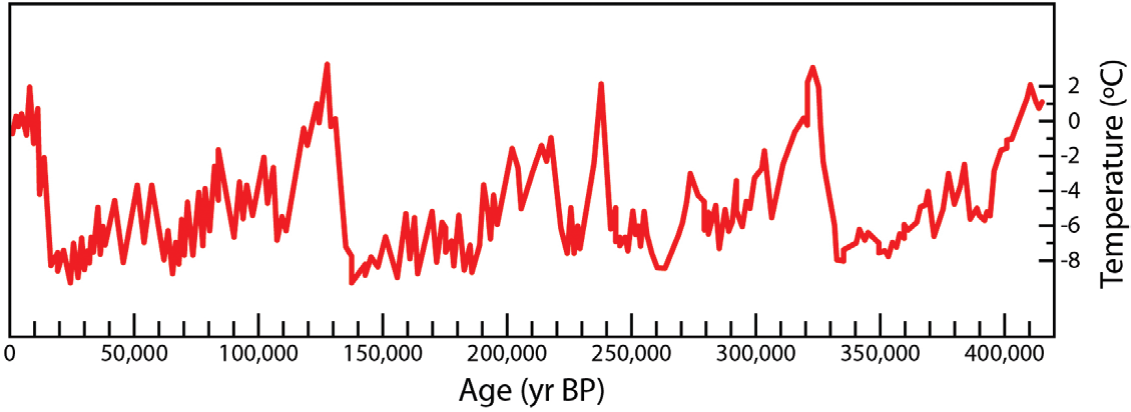
Record of isotopic temperature changes of the atmosphere extracted from an Antarctic ice core (Petit *et al.*, 1999). BP, Before present.

Throughout the course of evolution plants have been constantly confronted with changing environmental conditions, forcing them to adapt in order to survive. These changing conditions included temperature fluctuations (Fig. 1), scarce water resources, and high UV radiation. Although it is extremely difficult to precisely determine the temporal dynamics of prehistoric climate changes, it is undeniable that climate change has accelerated in the past century (Murray, 1997; Petit *et al.*, 1999). The fact that anthropogenic greenhouse gas emissions considerably contribute to this acceleration raises the question of whether plants will be able to adapt to the imposed environmental stress conditions. It is possible that they will run out of time to develop appropriate responses to counteract the detrimental effects. However, assuming that this is not the first time that plants have faced such conditions, they may already have suitable molecular mechanisms at their disposal, generated during previous challenges, to withstand the foreseen unfavorable conditions of increased temperatures and water shortages.

The majority of plant studies focus only on plant responses toward abiotic stresses and disregard the fact that plants normally live in close association with a plethora of different microorganisms, such as bacteria, fungi, oomycetes, and protists, and that millions of years of co-evolution have led to the establishment of highly specialized ecosystems in which plants constantly interact with their surrounding communities of commensal, symbiotic, and pathogenic microorganisms. In the climate change context, symbiotic relationships are of particular interest as they are supposed to translate, or already have translated, into mutually advantageous associations that can provide important fitness improvements. The concept of a mutual coexistence between dissimilar organisms, referred to as symbiosis (from the ancient Greek *sumbíōsis*, ‘living together’), was first described by Heinrich Anton de Bary (1879). Later, the terms symbiosis, symbiont, and symbiote were further defined by Hertig *et al*. (1937). Since these early observations, our insight into symbiotic associations of plants has substantially advanced to indicate that plant–microbe interactions are important to the structure, function, and health of plant communities and that symbiotic fungi contribute to—and may be even responsible for—the adaptations of plants to environmental stresses (Rodriguez *et al.*, 2004).

Given that practically all plants are symbiotic with fungi, which depend either partially (endophytes) or entirely (mycorrhizae) on the interaction with their host plant, we asked ourselves the question of whether it would be potentially interesting to investigate symbiotic interactions in plant–fungal communities known to inhabit ecosystems in extreme environments. Such ‘extreme’ communities may allow us to identify patterns of crosstalk or molecular mechanisms that facilitate the creation of more sustainable agricultural systems with increased performance levels, either by harnessing identified molecular mechanisms or by soil microbiome engineering approaches. There are plenty of examples of mutualistic fungi that can confer stress tolerance upon plants. For example, a morphologically defined group of fungi within the class Ascomycota, the so-called dark septate endophytes, are well known as endosymbionts of numerous plant species, including crop plants (Jumpponen and Trappe, 1998; Andrade-Linares *et al.*, 2011). Intriguingly, Marco Molina-Montenegro and colleagues recently reported the symbiosis of two fungal endophytes, *Penicillium chrysogenum* and *Penicillium brevicompactum*, with *Colobanthus quitensis* (Caryophyllaceae) and *Deschampsia antarctica* (Poaceae), two plants that are found in the Antarctic region. It was demonstrated that the isolated root endophytes are able to increase plant performance under UV-B radiation. Moreover, the inoculation of lettuce with the isolated fungi was shown to significantly improve the ecophysiological performance and yield of the plant under both normal and drought conditions (Molina-Montenegro *et al.*, 2016; Ramos *et al.*, 2018). Another highly interesting example of an ‘extreme’ symbiosis comes from the isolation of a *Fusarium culmorum* strain that has been identified as an endophytic symbiont of dunegrass (*Leymus mollis*) collected from costal beach habitats. Remarkably, the fungal strain isolated from plants collected from this salt-stress environment was more efficient in conferring salt-stress tolerance to dunegrass and tomato plants than a *F. culmorum* isolate purchased from the American Type Culture Collection; this reflects an ecological phenomenon referred to as habitat-adapted symbiosis. This habitat-specific phenomenon is suggested to provide an intergenomic epigenetic mechanism for plant adaptation and survival in high-stress environments (Rodriguez *et al.*, 2008). We made a similar observation with a *Fusarium* sp. strain isolated from sea-blite (*Suaeda maritima*) collected from saline ponds along coastal plains in India (R. Oelmüller, unpublished results).

Possibly the most extensively studied endosymbiotic fungus from an extreme environment is *Serendipita indica* (formerly named *Piriformospora indica*). *S. indica* (Agaricomycetes, Basidiomycota) is an axenically cultivable, root-colonizing endophytic fungus that was first isolated from two xerophytic woody shrubs, *Ziziphus nummularia* and *Prosopis juliflora*, in the Thar desert in India (Verma *et al.*, 1998). Closely related endophytic species have been isolated from western European and Namibian Fabaceae, Poaceae, and Araceae (Weiß *et al*., 2011). The initially identified isolate of *S. indica* is deposited at the Deutsche Sammlung von Mikroorganismen und Zellkulturen (DSMZ), Braunschweig, Germany (DSM 11827). The fungus possesses exceptional ability to substantially promote plant growth and performance, especially under stress conditions, and an outstanding versatility to colonize a very broad range of plant species (Waller *et al.*, 2005; Jacobs *et al.*, 2011; Gill *et al.*, 2016; Su *et al.*, 2017). The latter feature suggests the existence of conserved molecular mechanism(s) that facilitate promiscuous host selection, which makes *S. indica* such an interesting microorganism to study and led to a rapid suggestion that it could be applied to increase plant production (Varma *et al.*, 1999). In this review, we will provide a summary on the current knowledge of the molecular and biochemical crosstalk between *S. indica* and its hosts, and reflect on the possibilities to apply this knowledge to improve agricultural productivity.

## The fungus *Serendipita indica*

Although the basidiomycete *S. indica* is classified as an endophyte, the fungus possesses many characteristics that are generally attributed to arbuscular mycorrhizal fungi (AMF), for example, its lifestyle maintaining hyphae outside the host root. Like AMF, *S. indica* is able to promote the growth of host plants and increase the resistance of colonized plants to fungal pathogens and various abiotic stresses (Harman, 2011; Abdelaziz *et al.*, 2019). Additionally, *S. indica* is known to affect the secondary metabolism of colonized host plants and to promote seed production by host plants, some of which are of economic importance (Waller *et al.*, 2005; Strehmel *et al.*, 2016). However, unlike AMF, *S. indica* can be easily cultivated (Oelmüller *et al.*, 2009) and is capable of colonizing the roots of the model dicot plant *Arabidopsis thaliana* (Peškan-Berghöfer *et al.*, 2004). In fact, *S. indica* belongs to the newly described family of Sebacinaceae and the new order Sebacinales (Agaricomycetes) (Weiss *et al.*, 2004, 2011; Qiang *et al.*, 2012*a*; Weiß *et al.*, 2016).

Owing to the great scientific interest in this mutualistic symbiont over the past two decades, great advances have been made with respect to the genomic assessment and manipulation of *S. indica*. As early as 2009, the group of Alga Zuccaro described a method for the stable genetic transformation of the fungus (Zuccaro *et al.*, 2009), and this was followed by a report on the deep genomic study of its 25 Mb genome (Zuccaro *et al.*, 2011). These experimental advances represent highly valuable tools for further investigation of the molecular mechanisms that allow the fungus to grow readily on diverse media and to colonize an extremely wide range of mono- and dicotyledonous plants.

## Establishment of symbiosis

The genomic study additionally revealed that root colonization by *S. indica* proceeds in two steps (Zuccaro *et al.*, 2011). The interaction begins with a short biotrophic phase, which is followed by a saprophytic phase in which the fungus feeds on dead host plant cells. During the initial phase of root colonization, the host plant responds with increased programmed cell death triggered by the simultaneous induction of endoplasmic reticulum stress and repression of the unfolded protein response. Moreover, it is hypothesized that the endoplasmic reticulum stress translates into the induction of a vacuolar processing enzyme/caspase 1-like activity-dependent vacuolar cell death program (Deshmukh *et al.*, 2006; Qiang *et al.*, 2012*b*). Furthermore, the host plant responds by secreting symbiosis-specific proteins, including proteins related to growth, development, biotic and abiotic stress responses, and mucilage (Thürich *et al.*, 2018). At the same time, plant innate immunity is suppressed by the manipulation of a handful of different plant hormone signaling pathways to overcome plant defenses and establish a compatible interaction between the fungus and the host (Schäfer *et al.*, 2009; Jacobs *et al.*, 2011). In particular, levels of abscisic acid (ABA), salicylic acid, and jasmonic acid, as well as jasmonoyl-l-isoleucine, are reported to substantially increase during very early phases of the interaction. The induction of stress- and defense-related hormones in the host plant can be triggered by chemical compounds released by the fungus before physical contact. It may be concluded that these transient metabolic reactions serve to prepare the plant for the symbiotic interaction. Notably, the plant response is not restricted to the root system, but spreads to aerial tissues as well. Moreover, the response is only short lived, as hormone levels return to normal levels after 6 days of co-cultivation (Vahabi *et al.*, 2015).

The exact role of phytohormones in the establishment of the symbiotic interaction is still a matter of debate (Vadassery *et al.*, 2008; Schäfer *et al.*, 2009; Lee *et al.*, 2011; Hilbert *et al.*, 2012). The broad host range of *S. indica* suggests that it evolved highly sophisticated colonization strategies, recruiting a similar or identical hormone signaling pathway from host plants in order to successfully establish a symbiotic relationship. However, recent work has cast doubts on this hypothesis of a simple reprogramming of the plant hormone-signaling machinery. A comprehensive study of the role of jasmonic acid and gibberellic acid during the colonization of seven different plant species with *S. indica* highlighted considerable differences in root colonization and plant hormone action, suggesting a high degree of species specificity in the establishment of symbiosis (Liu *et al.*, 2019).

**Fig. 2.**
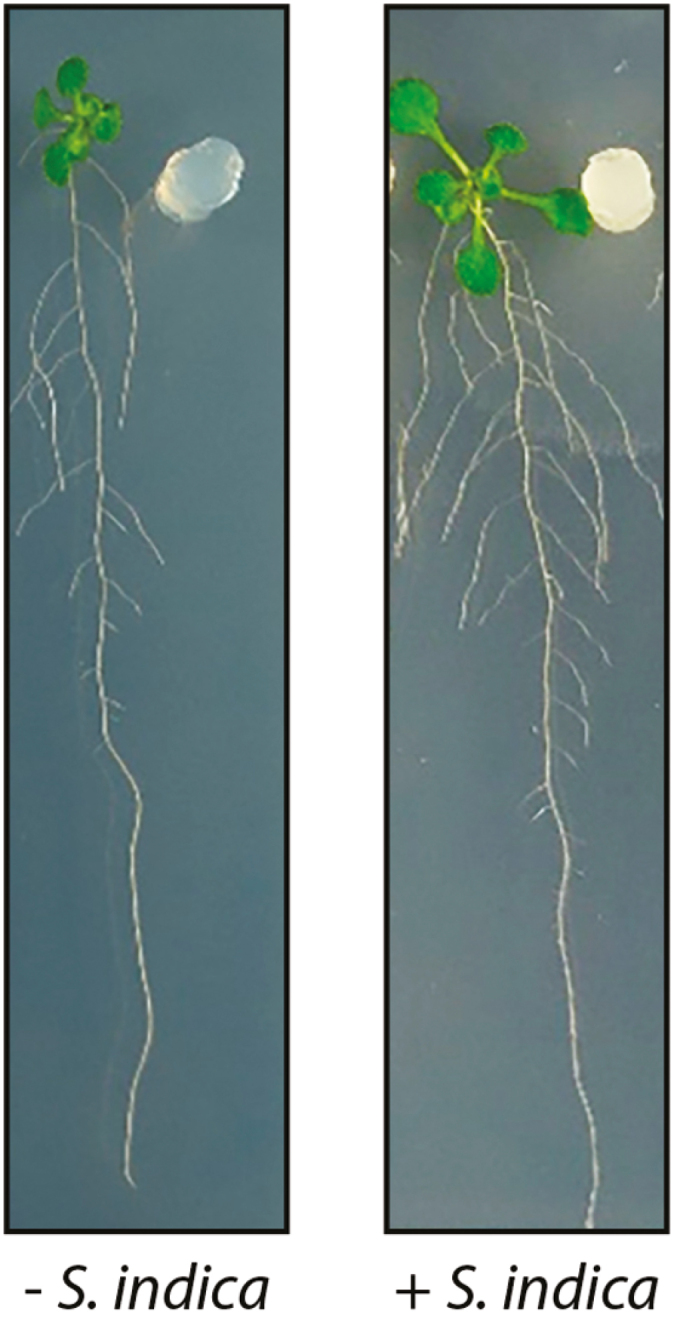
Growth-promoting effect of *S. indica* on young *A. thaliana* plants. The plants were grown either without *S. indica* (control, with a sterile media plug) or in the presence of a media plug that contained *S. indica* hyphae.

The controversial discussion regarding the contribution of auxins to fungus-mediated growth promotion and the establishment of the mutual interaction is also noteworthy. In particular, the root growth-promoting effect of *S. indica* implied the involvement of auxins, possibly the best-characterized class of plant hormones (Fig. 2). Auxins orchestrate virtually every aspect of plant growth and development (Davies, 2010). In plant roots, changes in local auxin levels cause a number of well-described phenotypes, including a dose-dependent increase in the length of epidermal-derived root hairs, a bimodal effect on primary root elongation, and a dose-dependent increase in the number of lateral root primordia (Overvoorde *et al.*, 2010). More importantly in this context, however, is the fact that indole-3-acetic acid (IAA) and several of its precursor molecules, such as indole-pyruvic acid (IPA), indole-3-acetamide (IAM), and tryptamine (TAM), are readily synthesized by various plant-interacting fungi and appear to contribute to fungal–plant interactions (Spaepen *et al.*, 2007; Fu *et al.*, 2015). *S. indica* was shown to synthesize IAA via IPA and indole-3-acetaldehyde (IAD), while other conceivable indolic intermediates, namely IAM, TAM, and indole-3-acetonitrile, were neither detected as endogenous compounds nor converted into IAA by the fungus (Hilbert *et al.*, 2012). Further experiments provided strong evidence that mycelium-derived IAA has no significant impact on growth promotion, but rather represents a compatibility factor that is important for the establishment of the biotrophic interaction (Hilbert *et al.*, 2012). More recently, however, another report demonstrated that *S. indica* hyphae contain considerable amounts of IAM and that both IAM and IAA levels increase during the colonization of *Brassica campestris* roots (Hua *et al.*, 2017). In any case, it seems that IAA levels in host plants temporarily increase during the early phases of the interaction, only to return to the levels of non-colonized roots shortly after (Vadassery *et al.*, 2008; Hilbert *et al.*, 2012). Genome-wide expression studies and gene ontology (GO) analyses did not provide clear-cut indications of a significant enrichment of genes listed in GO terms related to auxin biosynthesis or signaling during the first 14 days after infection (Lahrmann *et al.*, 2015). Interestingly, the content of conjugated auxin increases moderately over the course of colonization in infected roots (Vadassery *et al.*, 2008). This observation may suggest that the fungus either inactivates free IAA by itself or induces the expression of *Gretchen Hagen 3* (*GH3*) genes. *GH3* genes encode IAA-amidosynthases that catalyze the conjugation of free IAA to amino acids, thereby physiologically inactivating the plant hormone (Staswick *et al.*, 2005; Böttcher *et al.*, 2010, 2012). Remarkably, a recent study employing state-of-the-art live cell imaging techniques and liquid chromatography/mass spectrometry-based plant hormone analyses provided unequivocal evidence that the initial fungus-mediated increase of IAA in plant roots is seemingly adequate to induce lateral root formation within a very short timeframe (Meents *et al.*, 2019). In view of these findings, it appears tempting to speculate that the fungus-derived increase of IAA in the early phase of colonization is sufficient to trigger alterations in the developmental program of the host root, which result in morphological changes in the root architecture, facilitating the improved nutrition of the host plant through the extended root system in the long term.

**Fig. 3.**
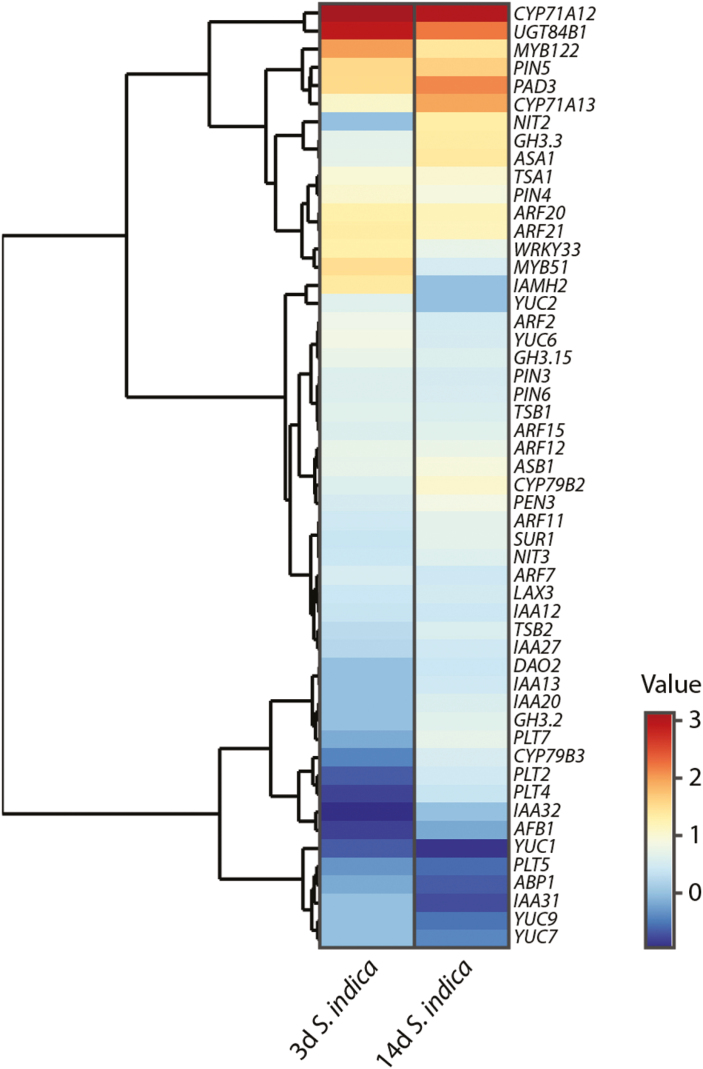
Hierarchical clustering analysis of auxin-related genes differentially regulated during the first 2 weeks of co-cultivation with *S. indica*. The gene expression levels were extracted from publicly available data (GSE60736, Lahrmann *et al.*, 2015) deposited in the Gene Expression Omnibus (GEO) repository for high-throughput microarray and next-generation sequence functional genomic datasets (Barrett *et al.*, 2013). A *P*-value of 0.05 after adjustment for multiple testing and log_2_ ratio >0.75 were arbitrarily chosen to select 45 differentially expressed genes in Arabidopsis seedlings co-cultivated with *S. indica* relative to mock-treated control plants.

A more detailed analysis of transcriptional changes of a group of approximately 140 genes related to auxin metabolism, transport, and signaling over the first 14 days of co-cultivation with *S. indica*, however, contradicts the notion that auxin-related genes are not considerably affected. The analysis revealed the induction of a set of *GH3* genes, namely *GH3.2*, *GH3.3*, and *GH3.15*, in the infected plants, which neatly matches the observed induction of IAA–amino acid conjugates (Vadassery *et al.*, 2008). Interestingly, the *UGT84B1* gene, which codes for a UDP-glycosyltransferase that has been described to catalyze the conjugation of free IAA to glucose (Jackson *et al.*, 2001, 2002), is also significantly induced. In line with these findings, it has been observed that infection with *S. indica* is sufficient to rescue the high-auxin phenotype of the *sur1-1* mutant through the reduction of free auxin levels (Vadassery *et al.*, 2008). Together, these experiments support the hypothesis that cellular auxin levels are increased and need to be actively intercepted by conjugation with either sugar or amino acids to prevent the over-accumulation of physiologically active free IAA. The latter assumption is further strengthened by the observed induction of *PIN5* expression. PIN5 is an auxin transporter that contributes to the maintenance of subcellular auxin homeostasis by mediating the flow of auxin from the cytoplasm into the lumen of the endoplasmic reticulum (Mravec *et al.*, 2009). Notably, two other *PIN* genes, *PIN3* and *PIN4*, also appear to be induced upon infection with *S. indica* (Fig. 3). While PIN3, together with auxin response factor 7 (ARF7), drives early steps in lateral root formation, PIN4 is known to be involved in the generation of auxin gradients and auxin canalization in root tips (Friml *et al.*, 2002; Chen *et al.*, 2015*b*; Laskowski and ten Tusscher, 2017). Thus, it is suggested that they are involved in triggering the root growth-promoting effect observed in *S. indica*-infected plants. A detailed study of the role of PIN3 and PIN4 in the infection process is currently under way in our laboratories.

**Fig. 4.**
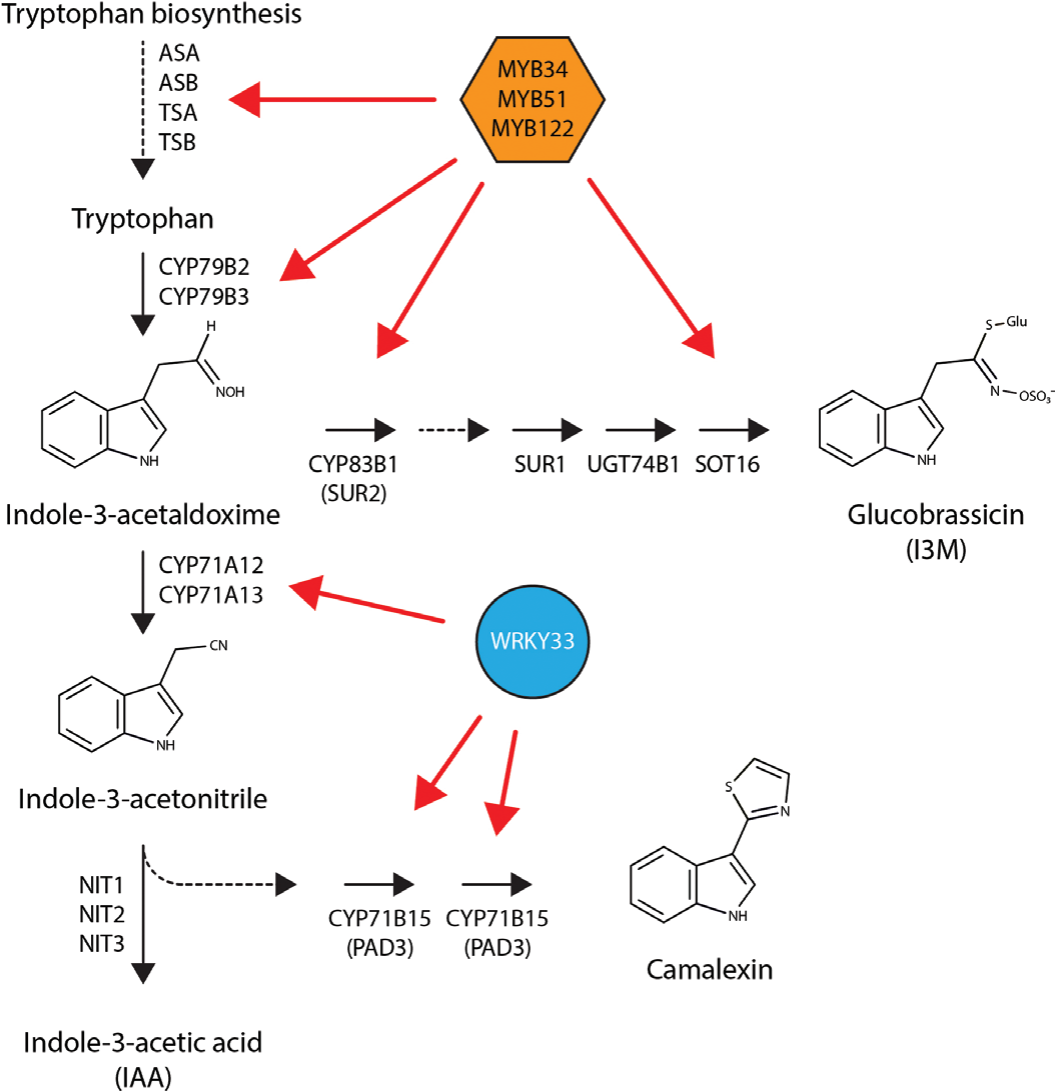
Regulation of the glucobrassicin and camalexin biosynthetic pathways by MYB and WRKY transcription factors. Red arrows indicate experimentally proven gene regulatory interactions (Birkenbihl *et al.*, 2012; Frerigmann and Gigolashvili, 2014; Frerigmann *et al.*, 2015). Dashed lines indicate multi-step reactions; in some cases, not all enzymes are yet known. ASA, ANTHRANILATE SYNTHASE α; ASB, ANTHRANILATE SYNTHASE β; CYP, cytochrome P450 enzymes; I3M, indole-3-methyl glucosinolate; NIT1–3, NITRILASE 1–3; PAD3, PHYTOALEXIN DEFICIENT 3; SUR1–2, SUPERROOT 1–2; SOT16, SULFOTRANSFERASE 16; TSA, TRYPTOPHAN SYNTHASE α; TSB, TRYPTOPHAN SYNTHASE β; UGT74B1, UDP-GLYCOSYLTRANSFERASE 74B1

Most notable, however, is the increased flux of metabolites into the production of the defense compounds camalexin and glucobrassicin. As can be seen in Fig. 3, a large proportion of the genes encoding relevant enzymes of the two corresponding biosynthetic pathways (Fig. 4) are significantly induced upon infection with *S. indica*. The biosynthesis of camalexin and glucobrassicin could be essential to limit colonization of the host plant with *S. indica*, thus allowing the beneficial interaction while preventing over-colonization (Nongbri *et al.*, 2012). The corresponding metabolic pathways are orchestrated by a small number of transcription factors, namely MYB34, MYB51, MYB122, and WRKY33 (Birkenbihl *et al.*, 2012; Frerigmann and Gigolashvili, 2014; Frerigmann *et al.*, 2015). Consistently, three out of the four transcription factors appear to be substantially induced over the course of infection. However, glucobrassicin and camalexin originate from a common precursor molecule, indole-3-acetaldoxime (IAOx), which is assumed to represent an intermediate in a *Brassica*-specific metabolic shunt (Pollmann *et al.*, 2006; Lehmann *et al.*, 2017). Although some recent publications have suggested that IAOx also occurs outside the Brassicaceae (Irmisch *et al*., 2013*a*, *b*; Luck *et al.*, 2016; Buezo *et al.*, 2019), a more general role for these compounds in the initial phase of the infection has to be doubted as long as a broader occurrence of IAOx in the plant kingdom is not unequivocally confirmed. Hence, metabolic engineering approaches on the basis of the IAOx shunt appear to offer only little prospect of improving agricultural productivity.

## The induction of cytosolic calcium in plant–fungus interactions

The rapid detection of specific external stimuli, as well as a timely and adequate response to them, is often instrumental to guarantee plant survival. Second messenger molecules, such as cyclic AMP or inositol triphosphate, are intracellular signaling molecules that are released by the cell in response to the perception of extracellular signals. They play important roles in the integration and transduction of signals, triggering physiological responses at the cellular level. Calcium (Ca^2+^) is a highly conserved and very versatile intracellular second messenger linking several extracellular cues with appropriate cellular responses, including growth and defense (Dodd *et al.*, 2010). In addition, Ca^2+^ is involved in providing tolerance to biotic and abiotic stresses (Kudla *et al.*, 2018). Owing to the functional conservation of Ca^2+^ signaling in plants, it is not at all surprising that the recruitment of Ca^2+^ signaling by a colonizing fungus represents another well-characterized mechanism that plays a key role in the establishment of plant–fungus interactions.

The interaction of microbes with plant roots often involves a type of chemical communication that commences with the recognition of chemical mediators at the cell surface of the host plant and engages sophisticated downstream plant immunity mechanisms that act as surveillance systems to detect the invasion of microbes into the host cell. In the recognition process, microbe-associated molecular patterns (MAMPs) or invasion patterns are perceived by specific receptors, which, in turn, activate pattern-triggered responses (van’t Padje *et al.*, 2016; Zipfel and Oldroyd, 2017). The response cascades that are triggered can either be suppressed, for example, by biotrophs, or employed, in the case of necrotrophs, to continue symbiosis (Cook *et al.*, 2015).

Cell wall extracts (CWEs) from *S. indica* have been described to promote the elevation of cytosolic Ca^2+^ concentration ([Ca^2+^]_cyt_), thereby mimicking the presence of the fungus and promoting plant growth in the initial phase of the interaction (Vadassery *et al.*, 2009). Intriguingly, this observation suggests that growth promotion can be uncoupled from the colonization of the root system with *S. indica* and that growth promotion depends on Ca^2+^. This is further supported by the fact that the CWE-mediated growth-promotion response can be blocked by the additional application of Ca^2+^-specific chelators such as BAPTA or LaCl_3_. However, it should be noted that even repeated treatment of roots with CWEs has not been sufficient to entirely replace the interaction with the endosymbiont (Vadassery *et al.*, 2009). Hence, it may be concluded that further factors or processes, apart from the perception of the active elicitor in the CWEs and the downstream Ca^2+^ signal, also play important roles in this context. The hydrolyzation of extracellular ATP (eATP) by specialized fungus-derived ecto-5′-nucleotidases and the resulting interference with the perception of eATP by the lectin receptor kinase DORN1 (Does not Respond to Nucleotides 1) could be such a missing process that apparently forms an integral part of the establishment of the symbiosis (Choi *et al.*, 2014*a*; Nizam *et al.*, 2019).

Mutant analyses revealed that CWE-mediated growth promotion in the *S. indica*–Arabidopsis interaction involves the activation of MITOGEN-ACTIVATED PROTEIN KINASE 6 (MAPK6), since the *mapk6* mutant shows no growth promotion upon treatment with CWEs. The Ca^2+^ influx-dependent activation of *MAPK6* is induced by numerous microbial elicitors (Nühse *et al.*, 2000; Lecourieux *et al.*, 2002) and represents a common theme in plant resistance to biotic stresses (Menke *et al.*, 2004; Takahashi *et al.*, 2007). Furthermore, camalexin biosynthesis is reported to be controlled through the MAPK3/MAPK6 cascade, activating WRKY33 through phosphorylation (Ren *et al.*, 2008; Li *et al.*, 2012), which functionally links the increase of [Ca^2+^]_cyt_ to the observed plant defense response upon *S. indica* colonization (Figs 3, 4).

Recent work identified the molecular nature of the active elicitor in *S. indica* CWEs. During root colonization, cellotriose (CT) is released by the fungus to initiate the symbiotic interaction of the fungus with the root (Johnson *et al.*, 2018). Plant roots perceive and respond to very low concentrations of short-chain β(1→4)-linked d-glucose units (cellooligomers). Cellooligomers are generally released after structural changes to the cell wall caused by either environmental or plant integral signals. They are suggested to give an account of the state and integrity of the cell wall, which facilitates the activation of appropriate local and distal responses (Souza *et al.*, 2017; Oelmüller, 2018).

The perception of CT by an as yet unidentified receptor results in a dose-dependent rapid and transient increase in [Ca^2+^]_cyt_, which induces mild defense responses including the induction of reactive oxygen species, changes in membrane potential, and the expression of genes associated with growth regulation and root development (Johnson *et al.*, 2018). CT perception is independent of BRI1-ASSOCIATED RECEPTOR KINASE 1 (BAK1), which is a well-known co-receptor contributing to the perception and integration of multiple MAMP-triggered responses, for instance, those toward bacterial flg22 or elf18 or fungal chitin, inducing [Ca^2+^]_cyt_ elevation and downstream immune responses (Lu *et al.*, 2010; Zhang *et al.*, 2010; Shi *et al.*, 2013; Li *et al.*, 2014; Kadota *et al.*, 2015). Moreover, mutants of the proposed Ca^2+^ channels GLU-LIKE RECEPTOR (GLR) 2.4, GLR2.5, GLR3.3, and the vacuolar TWO PORE CHANNEL 1 (TPC1) were not impaired in CT signaling. This strongly suggests that they are also not involved in the process, although they have previously been reported to contribute to wound signaling and wounding-induced systemic Ca^2+^ elevations (Peiter *et al.*, 2005; Li *et al.*, 2013; Manzoor *et al.*, 2013; Choi *et al.*, 2014*b*; Kiep *et al.*, 2015). However, the mechanisms that are involved in the rapid Ca^2+^ influx have yet to be identified.

In addition to the question of how the Ca^2+^ influx is brought about on the molecular level, the complexity of Ca^2+^ signaling, involving a large number of different Ca^2+^-binding proteins to specifically decode incoming Ca^2+^ signals, makes it tremendously difficult to address the question of which molecular components contribute to the integration of the Ca^2+^ signals induced during *S. indica* infection. Complicating the situation even further, different Ca^2+^ signatures appear to trigger a variety of functions via the signature-specific activation of corresponding Ca^2+^-sensor proteins (McAinsh and Pittman, 2009; Johnson *et al.*, 2011). All Ca^2+^-sensor proteins, however, contain at least one so-called EF-hand motif (helix-loop-helix domain) in their primary sequence, which mediates the binding of Ca^2+^ to the sensor. The Arabidopsis proteome contains approximately 250 proteins with EF-hand motifs, and at least 100 have been classified as Ca^2+^-sensor proteins (Day *et al.*, 2002; Hashimoto and Kudla, 2011). With respect to the nature of their response domain, which can be either a kinase or a transcription regulation domain, sensor proteins are further divided into sensor relay proteins or sensor responders. The latter class of sensors are particularly versatile, as they combine a sensor and response domain in a single protein and can thus directly transduce the Ca^2+^ signal to downstream target proteins by phosphorylation. CALCIUM-DEPENDENT PROTEIN KINASEs (CDPKs) represent an important family of these sensor responder proteins, and 34 members of this family have been identified in Arabidopsis (Cheng *et al.*, 2002). CDPKs have been demonstrated to be activated by Ca^2+^ signals during the course of interactions of plant roots with biotrophic microbes. They are speculated to control host entry and accommodation in the plant through the efficient suppression of corresponding plant defense responses (Freymark *et al.*, 2007; Chen *et al.*, 2015*a*). Recent work has suggested the involvement of CDPKs in the *S. indica*–plant interaction as well, as *S. indica* CWE-mediated [Ca^2+^]_cyt_ elevation has been shown to promote tuberization in potato via a pathway that involves the potato CDPK1 sensor responder (Upadhyaya *et al.*, 2013). The work by Johnson *et al*. (2018) supports this hypothesis, because their microarray data provide strong evidence for the induction of a number of CDPK proteins upon CT treatment. A more detailed analysis of CDPKs involved in the establishment and maintenance of the fungus–plant symbiosis has yet to be carried out, although it might be very difficult to achieve this task owing to the large number of CDPK family members in plants and their presumably partially overlapping functions. Available microarray data also revealed the differential expression of a small number of sensor relay proteins—CALMODULIN-LIKE 37 (CML37) and CML38 as well as CBL-INTERACTING PROTEIN KINASE 13 (CIPK13) and CIPK20—but their role in fungus–plant interaction is even less well investigated and remains a matter of debate (Vahabi *et al.*, 2015; Johnson *et al.*, 2018).

## *Serendipita indica* and the growth–defense tradeoff enigma

The initial phase of root colonization by *S. indica* involves the mounting of defense responses by the host plant (Zuccaro *et al.*, 2011; Vahabi *et al.*, 2015; Johnson *et al.*, 2018). Such defense responses frequently come at the cost of a substantial reduction in growth and reproduction, which carries important implications for agriculture. However, despite the obvious importance of this tradeoff between growth and defense in shaping plant productivity in agricultural ecosystems, the molecular mechanisms that connect plant growth with plant defense responses are only poorly understood. From the classical point of view, plants employ physiological tradeoffs to allocate their limited metabolic resources between the generation of defense-related protective compounds on the one hand and associated morphological structures on the other hand. In other words, plants face a dilemma: to decide whether to grow or to defend themselves (Herms and Mattson, 1992; Züst *et al.*, 2015). Although most ecological studies of plant resistance to herbivores or pathogens still use the concept of growth–defense tradeoffs as their major paradigm (Denancé *et al.*, 2013; Huot *et al.*, 2014), more recent studies have questioned this simplistic view (Kliebenstein, 2016; Schuman and Baldwin, 2016). They point out that growth inhibition in response to, for example, herbivory is likely not the result of the simple channeling of photoassimilates from growth to defense but rather due to a conserved transcriptional network that serves the purpose of attenuating growth upon wounding (Campos *et al.*, 2016).

However, whichever point of view is chosen, it seems as if plant defense is always realized at the expense of growth, whether through the reallocation of metabolic resources or the activation of specific gene regulatory networks hardwired to confront biotic stress situations. When inspecting the beneficial effect of the co-cultivation of plants with *S. indica* it becomes obvious that there is a discrepancy between the observed effects and the classical growth–defense tradeoff concept. Although *S. indica* infection triggers defense responses, plant biomass production and productivity in terms of seed yield are increased (Oelmüller *et al.*, 2009; Achatz *et al.*, 2010). It therefore has to be concluded that, along with the basic initial plant defense response, further mechanisms are triggered by the fungus, allowing the host plant to grow despite all of the metabolic restrictions. In order to sustain growth without paying the price of increased stress susceptibility, the underlying mechanisms likely involve the improvement of plant nutrition, which is essential to mitigate the provoked metabolic deficit. The pronounced extension of the root system of infected plants (Fig. 2) suggests that the penetration and exploration of new areas of soil plays an important role in this context.

In their natural soil habitats, plants interact with a broad variety of microorganisms. In particular, microbes in the rhizosphere play important roles in the acquisition of soil nutrients, including the most important macronutrients, nitrogen (N) and phosphorus (P) (Hayat *et al.*, 2010; Hacquard *et al.*, 2015, 2017). Soil microbes can increase nutrient uptake by converting insoluble complexes, which represent unavailable forms of nutrients in the soil, to their ionic forms, which are more suitable for assimilation via the roots. In addition, the beneficial relationships of AMFs with their host plants have been extensively studied. The mycorrhizal symbiosis is known to facilitate improved access to soil nutrients, particularly phosphate (Chiu and Paszkowski, 2019). As previously pointed out, *S. indica* and other Sebacinales resemble AMFs in many aspects. Thus, it does not appear improbable that those mutualistic fungi may also impact nutrient uptake in their host plants, and indeed similar effects have been reported (Malla *et al.*, 2004; Shahollari *et al.*, 2005; Saddique *et al.*, 2018). In mycorrhizal communities, nutrient exchange involves a number of specific transporters for both the uptake of nutrients by the fungus and their subsequent exchange with the host (Wang *et al.*, 2017). For most other beneficial endophytic fungi, including *S. indica*, the molecular mechanisms involved in nutrient exchange are only poorly understood and need to be further investigated. Although it is generally assumed that an effective mechanism exists for nutrient absorption and translocation to the host plant by *S. indica*, in exchange for plant-derived carbon sources (Khalid *et al.*, 2019), the translocation of inorganic phosphate by *S. indica* is controversial. *S. indica* has been reported to contain a high-affinity phosphate transporter (PiPT) that is presumably involved in the transfer of inorganic phosphate to the host plant (Yadav *et al.*, 2010; Kumar *et al.*, 2011). However a more recent study claimed that *S. indica* interferes primarily with P_i_ distribution and metabolism, rather than directly promoting phosphate uptake from the soil (Bakshi *et al.*, 2017).

With respect to N, it has been suggested that the inoculation of Arabidopsis and *Nicotiana tabacum* with *S. indica* induces N uptake and translocation from the culture medium to the aerial parts of the plant. The reported induction of N uptake is seemingly linked to the stimulation of nitrate reductase activity, a key step in nitrate assimilation, and the transcriptional activation of *NITRATE REDUCTASE 2* (*NIA2*) expression in Arabidopsis (Sherameti *et al.*, 2005). In summary, the available data support the idea that *S. indica* encroaches on the primary metabolism in host plant roots by delivering nutrients necessary for increased growth and development. The exact mechanisms by which *S. indica* achieves this, however, remain largely unknown.

## Induced drought and salt resistance

In the global climate change scenario, plant growth and productivity are greatly affected by drought stress, and plants have to adapt to prevailing unfavorable conditions in order to survive (Shinozaki and Yamaguchi-Shinozaki, 2007). Drought provokes a number of interconnected physiological and biochemical responses, including stomatal closure, repression of plant growth and photosynthetic activity, and activation of respiration (Schroeder *et al.*, 2001; Flexas *et al.*, 2004; Roelfsema and Hedrich, 2005; Rennenberg *et al.*, 2006). Work conducted over recent years has identified a large number of drought-inducible genes, which can be divided into two major groups: (i) genes that encode proteins directly involved in conveying abiotic stress tolerance, and (ii) genes encoding regulatory proteins, which interfere with signal transduction or the expression of stress-responsive genes. ABA is known to play a central role in the conversion of abiotic stress signals into appropriate cellular responses, although some ABA-independent signaling pathways have also been shown to contribute to the transcriptional control of abiotic stress-response genes.

*S. indica* was initially isolated from the roots of shrubs growing in the Thar desert in India. Hence, it is not surprising that this endosymbiont is also capable of attenuating the negative effects of drought stress effects in its host plant. Experiments with Arabidopsis, rice, and maize revealed that plants inoculated with *S. indica* performed considerably better under drought conditions (Sherameti *et al.*, 2008; Hosseini *et al.*, 2018; Saddique *et al.*, 2018; Zhang *et al.*, 2018). Microarray analyses performed by Zhang *et al.* (2018) indicated that a quite diverse set of stress-related genes (up to 2037 genes after 12 h of drought stress treatment) responded differentially in plants co-cultivated with *S. indica*. The detailed breakdown of the transcriptomic data revealed that *S. indica* promotes maize root growth under drought stress conditions largely through the stimulation of microtubular processes and the strengthening of the plant’s redox capacity by adjusting its carbon–sulfur balance.

Apart from drought stress, soil salinity represents an increasingly serious environmental threat that affects plant growth and yield. Soil salinity disturbs nutrient absorption by the roots, leading to osmotic and ionic imbalances and oxidative damage (Flowers, 2004; Ruiz-Lozano *et al.*, 2012). Similar to drought stress, salt stress leads to stomatal closure because it reduces the water potential of leaves, which results in decreased photosynthetic activity and increased photodamage (Baker and Rosenqvist, 2004). Several studies have indicated that *S. indica* is able to increase plant tolerance to salinity, partly through the increased conservation of photosynthetic pigments to reduce photodamage (Waller *et al.*, 2005; Jogawat *et al.*, 2013; Sharma *et al.*, 2014; Ghorbani *et al.*, 2018). However, the precise mechanism by which *S. indica* improves plant growth under conditions of salt stress has yet to be elucidated. The most recent studies underpin the idea that *S. indica* colonization of roots improves potassium (K^+^)/sodium (Na^+^) homeostasis through the transcriptional regulation of the cyclic nucleotide-gated channel *CNGC15* and plant cation/proton antiporter (*NHX*) genes, including *SOS1*. In addition, the water uptake potential of host plant roots is increased by the transcriptional regulation of aquaporins, in particular PIPs (plasma membrane intrinsic proteins) and TIPs (tonoplast intrinsic proteins), which have been suggested to be involved in water use efficiency and the maintenance of water equilibrium in plants subjected to diverse environmental stresses (Sade *et al.*, 2010; Ghorbani *et al.*, 2019). Intriguingly, the ion homeostasis- and water status-related effects triggered in the host plant are accompanied by an active Na^+^ detoxification of plant cells by *S. indica*. The reduction of Na^+^ contents in plants colonized with *S. indica* under saline conditions has been attributed to the induction of two ENA ATPases, *SiENA1* and *SiENA5*, which are involved in K^+^/Na^+^ and Na^+^ efflux, respectively (Lanza *et al.*, 2019). In consequence, this active Na^+^ detoxification suggests the existence of a barrier effect evoked by *S. indica* that prevents the accumulation of cations in the plant root. Taken together, recent work sheds some light on the molecular bases and mechanisms of *S. indica*-mediated improved salt tolerance. Nonetheless, there are still significant gaps in our current understanding of the processes involved, most importantly concerning the underlying gene regulatory networks.

## Prospects and conclusions

Fungi of the order Sebacinales have been identified all around the world, including in extreme ecosystems such as deserts. The root endophyte *S. indica* is capable of associating with all plants tested so far, transferring growth benefits and increased stress tolerance to its host plants under a broad range of different climate, temperature, and growth conditions. As is evident from the data discussed in this review, the molecular mechanisms that confer the beneficial effects upon host plants are highly complex and multi-layered, which makes it unlikely that a single master regulator will be identified that could be used in biotechnological approaches to unleash the full repertoire of fungal effects exerted during symbiosis in transgenic plants. The increasing insight into the molecular bases of the plant–fungal symbiosis will most probably provide evidence for suitable target genes that drive isolated facets of the interaction, such as growth promotion or increased biotic and abiotic stress tolerance.

A particularly interesting starting point for the improvement of agricultural productivity using information gained from studies of plant–microbe interactions is provided by the identification of the cellooligomer CT, which is the active elicitor of [Ca^2+^]_cyt_ elevations in *S. indica* CWEs. Apart from the induction of diverse defense responses, cellular Ca^2+^ signatures have recently been reported to directly contribute to primary root growth and development (Leitão *et al.*, 2019). However, the application of CT does not provide the full effect of symbiosis, and additionally CT is far too expensive for application in the field to be cost-effective. Nevertheless, some more investigation in this direction, for example, by taking a chemical genetics approach to identify reagents that trigger similar physiological responses and can be applied to soils as plant biostimulants to improve plant productivity, would be valuable. Subsequent studies should include a comprehensive examination of the general applicability of identified compounds and the best way to formulate them. A formulation employing slowly degrading coated beads that persist over a long period of time, as is used for enhanced-efficiency fertilizers, could be an effective way to apply putative CT-like reagents (Timilsena *et al.*, 2015). By using such a formulation, the micro-dosage present in the symbiosis could be emulated. The biochemical nature of the compound(s) also has to be considered. CT itself is an energy-rich carbon source that can most likely be used by other saprophytic fungi present in the soil that are able to degrade the triose. In this respect, a slow-release formulation could also help to contain the growth of unwanted fungal pathogens through limiting its availability as a source of carbon. At best, the chemical genetics approach may provide evidence for chemical compounds with CT-like effects on crops that are not digestible by microbes and, therefore, do not represent an easily accessible carbon source. However, with respect to organic farming practices in particular, the application of synthetic compounds to the field does not come without disadvantages.

In conclusion, the direct application of the fungus *S. indica* as a biostimulant is currently possibly the most suitable method to make use of the beneficial traits transferred by endophytic fungi to their host plants. *S. indica* offers the huge advantage that it will grow axenically and without a host plant. Moreover, it can be propagated at large scale and used as a biocontrol agent (Sun *et al.*, 2014), which underlines its high potential for biotechnological and agricultural applications.

## References

[CIT0001] Abdelaziz ME, Abdelsattar M, Abdeldaym EA, Atia MAM, Mahmoud AWM, Saad MM, Hirt H 2019 *Piriformospora indica* alters Na^+^/K^+^ homeostasis, antioxidant enzymes and *LeNHX1* expression of greenhouse tomato grown under salt stress. Scientia Horticulturae 256, 108532.

[CIT0002] Achatz B, Kogel KH, Franken P, Waller F 2010 *Piriformospora indica* mycorrhization increases grain yield by accelerating early development of barley plants. Plant Signaling & Behavior 5, 1685–1687. 21150264 10.4161/psb.5.12.14112 PMC3115137

[CIT0003] Aglawe SB, Barbadikar KM, Mangrauthia SK, Madhav MS 2018 New breeding technique “genome editing” for crop improvement: applications, potentials and challenges. 3 Biotech 8, 336. 10.1007/s13205-018-1355-3 PMC6056351 30073121

[CIT0004] Andrade-Linares DR, Grosch R, Franken P, Rexer KH, Kost G, Restrepo S, de Garcia MC, Maximova E 2011 Colonization of roots of cultivated *Solanum lycopersicum* by dark septate and other ascomycetous endophytes. Mycologia 103, 710–721. 21307164 10.3852/10-329

[CIT0005] Baker NR, Rosenqvist E 2004 Applications of chlorophyll fluorescence can improve crop production strategies: an examination of future possibilities. Journal of Experimental Botany 55, 1607–1621. 15258166 10.1093/jxb/erh196

[CIT0006] Bakshi M, Sherameti I, Meichsner D, Thürich J, Varma A, Johri AK, Yeh KW, Oelmüller R 2017 *Piriformospora indica* reprograms gene expression in Arabidopsis phosphate metabolism mutants but does not compensate for phosphate limitation. Frontiers in Microbiology 8, 1262. 28747898 10.3389/fmicb.2017.01262 PMC5506084

[CIT0007] Barrett T, Wilhite SE, Ledoux P, et al 2013 NCBI GEO: archive for functional genomics data sets–update. Nucleic Acids Research 41, D991–D995. 23193258 10.1093/nar/gks1193 PMC3531084

[CIT0008] Birkenbihl RP, Diezel C, Somssich IE 2012 Arabidopsis WRKY33 is a key transcriptional regulator of hormonal and metabolic responses toward *Botrytis cinerea* infection. Plant Physiology 159, 266–285. 22392279 10.1104/pp.111.192641 PMC3375964

[CIT0009] Böttcher C, Dennis EG, Booker GW, Polyak SW, Boss PK, Davies C 2012 A novel tool for studying auxin-metabolism: the inhibition of grapevine indole-3-acetic acid-amido synthetases by a reaction intermediate analogue. PLoS One 7, e37632. 22649546 10.1371/journal.pone.0037632 PMC3359377

[CIT0010] Böttcher C, Keyzers RA, Boss PK, Davies C 2010 Sequestration of auxin by the indole-3-acetic acid-amido synthetase GH3-1 in grape berry (*Vitis vinifera* L.) and the proposed role of auxin conjugation during ripening. Journal of Experimental Botany 61, 3615–3625. 20581124 10.1093/jxb/erq174

[CIT0011] Buezo J, Esteban R, Cornejo A, López-Gómez P, Marino D, Chamizo-Ampudia A, Gil MJ, Martínez-Merino V, Moran JF 2019 IAOx induces the SUR phenotype and differential signalling from IAA under different types of nitrogen nutrition in *Medicago truncatula* roots. Plant Science 287, 110176. 31481210 10.1016/j.plantsci.2019.110176

[CIT0012] Campos ML, Yoshida Y, Major IT, et al 2016 Rewiring of jasmonate and phytochrome B signalling uncouples plant growth-defense tradeoffs. Nature Communications 7, 12570. 10.1038/ncomms12570 PMC5155487 27573094

[CIT0013] Chen J, Gutjahr C, Bleckmann A, Dresselhaus T 2015a Calcium signaling during reproduction and biotrophic fungal interactions in plants. Molecular Plant 8, 595–611. 25660409 10.1016/j.molp.2015.01.023

[CIT0014] Chen Q, Liu Y, Maere S, et al 2015b A coherent transcriptional feed-forward motif model for mediating auxin-sensitive *PIN3* expression during lateral root development. Nature Communications 6, 8821. 10.1038/ncomms9821 PMC4673502 26578065

[CIT0015] Cheng SH, Willmann MR, Chen HC, Sheen J 2002 Calcium signaling through protein kinases. The Arabidopsis calcium-dependent protein kinase gene family. Plant Physiology 129, 469–485. 12068094 10.1104/pp.005645 PMC1540234

[CIT0016] Chiu CH, Paszkowski U 2019 Mechanisms and impact of symbiotic phosphate acquisition. Cold Spring Harbor Perspectives in Biology 11, a034603. 30910773 10.1101/cshperspect.a034603 PMC6546048

[CIT0017] Choi J, Tanaka K, Cao Y, Qi Y, Qiu J, Liang Y, Lee SY, Stacey G 2014a Identification of a plant receptor for extracellular ATP. Science 343, 290–294. 24436418 10.1126/science.343.6168.290

[CIT0018] Choi WG, Toyota M, Kim SH, Hilleary R, Gilroy S 2014b Salt stress-induced Ca^2+^ waves are associated with rapid, long-distance root-to-shoot signaling in plants. Proceedings of the National Academy of Sciences, USA 111, 6497–6502. 10.1073/pnas.1319955111 PMC4035928 24706854

[CIT0019] Cook DE, Mesarich CH, Thomma BP 2015 Understanding plant immunity as a surveillance system to detect invasion. Annual Review of Phytopathology 53, 541–563. 10.1146/annurev-phyto-080614-120114 26047564

[CIT0020] Davies PJ 2010 Plant hormones. biosynthesis, signal transduction, action! Dordrecht: Springer Netherlands.

[CIT0021] Day IS, Reddy VS, Shad Ali G, Reddy AS 2002 Analysis of EF-hand-containing proteins in *Arabidopsis*. Genome Biology 3, RESEARCH0056. 12372144 10.1186/gb-2002-3-10-research0056 PMC134623

[CIT0022] de Bary A 1879 Die Erscheinung der Symbiose. Vortrag auf der Versammlung der Naturforscher und Ärtze zu Cassel. Strassburg: Verlag von K. J. Trübner, 1–30.

[CIT0023] Denancé N, Sánchez-Vallet A, Goffner D, Molina A 2013 Disease resistance or growth: the role of plant hormones in balancing immune responses and fitness costs. Frontiers in Plant Science 4, 155. 23745126 10.3389/fpls.2013.00155 PMC3662895

[CIT0024] Deshmukh S, Hückelhoven R, Schäfer P, Imani J, Sharma M, Weiss M, Waller F, Kogel KH 2006 The root endophytic fungus *Piriformospora indica* requires host cell death for proliferation during mutualistic symbiosis with barley. Proceedings of the National Academy of Sciences, USA 103, 18450–18457. 10.1073/pnas.0605697103 PMC1697795 17116870

[CIT0025] Dodd AN, Kudla J, Sanders D 2010 The language of calcium signaling. Annual Review of Plant Biology 61, 593–620. 10.1146/annurev-arplant-070109-104628 20192754

[CIT0026] FAO, IFAD, WFP 2015 The state of food insecurity in the world 2015. Rome: Food and Agriculture Organization of the United Nations.

[CIT0027] Fedoroff NV 2015 Food in a future of 10 billion. Agriculture & Food Security 4, 11.

[CIT0028] Flexas J, Bota J, Loreto F, Cornic G, Sharkey TD 2004 Diffusive and metabolic limitations to photosynthesis under drought and salinity in C_3_ plants. Plant Biology 6, 269–279. 15143435 10.1055/s-2004-820867

[CIT0029] Flowers TJ 2004 Improving crop salt tolerance. Journal of Experimental Botany 55, 307–319. 14718494 10.1093/jxb/erh003

[CIT0030] Frerigmann H, Gigolashvili T 2014 MYB34, MYB51, and MYB122 distinctly regulate indolic glucosinolate biosynthesis in *Arabidopsis thaliana*. Molecular Plant 7, 814–828. 24431192 10.1093/mp/ssu004

[CIT0031] Frerigmann H, Glawischnig E, Gigolashvili T 2015 The role of MYB34, MYB51 and MYB122 in the regulation of camalexin biosynthesis in *Arabidopsis thaliana*. Frontiers in Plant Science 6, 654. 26379682 10.3389/fpls.2015.00654 PMC4548095

[CIT0032] Freymark G, Diehl T, Miklis M, Romeis T, Panstruga R 2007 Antagonistic control of powdery mildew host cell entry by barley calcium-dependent protein kinases (CDPKs). Molecular Plant-Microbe Interactions 20, 1213–1221. 17918623 10.1094/MPMI-20-10-1213

[CIT0033] Friml J, Benková E, Blilou I, et al 2002 AtPIN4 mediates sink-driven auxin gradients and root patterning in *Arabidopsis*. Cell 108, 661–673. 11893337 10.1016/s0092-8674(02)00656-6

[CIT0034] Fu SF, Wei JY, Chen HW, Liu YY, Lu HY, Chou JY 2015 Indole-3-acetic acid: a widespread physiological code in interactions of fungi with other organisms. Plant Signaling & Behavior 10, e1048052. 26179718 10.1080/15592324.2015.1048052 PMC4623019

[CIT0035] Gartland KMA, Gartland JS 2018 Opportunities in biotechnology. Journal of Biotechnology 282, 38–45. 29890193 10.1016/j.jbiotec.2018.06.303

[CIT0036] Ghorbani A, Omran VOG, Razavi SM, Pirdashti H, Ranjbar M 2019 *Piriformospora indica* confers salinity tolerance on tomato (*Lycopersicon esculentum* Mill.) through amelioration of nutrient accumulation, K^+^/Na^+^ homeostasis and water status. Plant Cell Reports 38, 1151–1163. 31152194 10.1007/s00299-019-02434-w

[CIT0037] Ghorbani A, Razavi SM, Ghasemi Omran VO, Pirdashti H 2018 *Piriformospora indica* inoculation alleviates the adverse effect of NaCl stress on growth, gas exchange and chlorophyll fluorescence in tomato (*Solanum lycopersicum* L.). Plant Biology 20, 729–736. 29575688 10.1111/plb.12717

[CIT0038] Gill SS, Gill R, Trivedi DK, et al 2016 *Piriformospora indica*: potential and significance in plant stress tolerance. Frontiers in Microbiology 7, 332. 27047458 10.3389/fmicb.2016.00332 PMC4801890

[CIT0039] Hacquard S, Garrido-Oter R, González A, et al 2015 Microbiota and host nutrition across plant and animal kingdoms. Cell Host & Microbe 17, 603–616. 25974302 10.1016/j.chom.2015.04.009

[CIT0040] Hacquard S, Spaepen S, Garrido-Oter R, Schulze-Lefert P 2017 Interplay between innate immunity and the plant microbiota. Annual Review of Phytopathology 55, 565–589. 10.1146/annurev-phyto-080516-035623 28645232

[CIT0041] Harman GE 2011 Multifunctional fungal plant symbionts: new tools to enhance plant growth and productivity. New Phytologist 189, 647–649. 21223281 10.1111/j.1469-8137.2010.03614.x

[CIT0042] Hashimoto K, Kudla J 2011 Calcium decoding mechanisms in plants. Biochimie 93, 2054–2059. 21658427 10.1016/j.biochi.2011.05.019

[CIT0043] Hayat R, Ali S, Amara U, Khalid R, Ahmed I 2010 Soil beneficial bacteria and their role in plant growth promotion: a review. Annals of Microbiology 60, 579–598.

[CIT0044] Herms DA, Mattson WJ 1992 The dilemma of plants: to grow or defend. Quarterly Review of Biology 67, 283–335.

[CIT0045] Hertig M, Taliaferro WH, Schwartz B 1937 The terms symbiosis, symbiont and symbiote. Journal of Parasitology 23, 326–329.

[CIT0046] Hilbert M, Voll LM, Ding Y, Hofmann J, Sharma M, Zuccaro A 2012 Indole derivative production by the root endophyte *Piriformospora indica* is not required for growth promotion but for biotrophic colonization of barley roots. New Phytologist 196, 520–534. 22924530 10.1111/j.1469-8137.2012.04275.x

[CIT0047] Hosseini F, Mosaddeghi MR, Dexter AR, Sepehri M 2018 Maize water status and physiological traits as affected by root endophytic fungus *Piriformospora indica* under combined drought and mechanical stresses. Planta 247, 1229–1245. 29453661 10.1007/s00425-018-2861-6

[CIT0048] Hua MD, Senthil Kumar R, Shyur LF, Cheng YB, Tian Z, Oelmüller R, Yeh KW 2017 Metabolomic compounds identified in *Piriformospora indica*-colonized Chinese cabbage roots delineate symbiotic functions of the interaction. Scientific Reports 7, 9291. 28839213 10.1038/s41598-017-08715-2 PMC5571224

[CIT0049] Huot B, Yao J, Montgomery BL, He SY 2014 Growth–defense tradeoffs in plants: a balancing act to optimize fitness. Molecular Plant 7, 1267–1287. 24777989 10.1093/mp/ssu049 PMC4168297

[CIT0050] Irmisch S, McCormick AC, Boeckler GA, et al 2013a Two herbivore-induced cytochrome P450 enzymes CYP79D6 and CYP79D7 catalyze the formation of volatile aldoximes involved in poplar defense. The Plant Cell 25, 4737–4754. 24220631 10.1105/tpc.113.118265 PMC3875747

[CIT0051] Irmisch S, Unsicker SB, Gershenzon J, Köllner TG 2013b Identification and characterization of CYP79D6v4, a cytochrome P450 enzyme producing aldoximes in black poplar (*Populus nigra*). Plant Signaling & Behavior 8, e27640. 24390071 10.4161/psb.27640 PMC4091388

[CIT0052] ISAAA 2017 Pocket K No. 4: GM crops and the environment. Los Baños, Laguna, International Service for the Acquisition of Agri-biotech Applications.

[CIT0053] Jackson RG, Kowalczyk M, Li Y, Higgins G, Ross J, Sandberg G, Bowles DJ 2002 Over-expression of an *Arabidopsis* gene encoding a glucosyltransferase of indole-3-acetic acid: phenotypic characterisation of transgenic lines. The Plant Journal 32, 573–583. 12445128 10.1046/j.1365-313x.2002.01445.x

[CIT0054] Jackson RG, Lim EK, Li Y, Kowalczyk M, Sandberg G, Hoggett J, Ashford DA, Bowles DJ 2001 Identification and biochemical characterization of an *Arabidopsis* indole-3-acetic acid glucosyltransferase. Journal of Biological Chemistry 276, 4350–4356. 11042207 10.1074/jbc.M006185200

[CIT0055] Jacobs S, Zechmann B, Molitor A, Trujillo M, Petutschnig E, Lipka V, Likpa V, Kogel KH, Schäfer P 2011 Broad-spectrum suppression of innate immunity is required for colonization of Arabidopsis roots by the fungus *Piriformospora indica*. Plant Physiology 156, 726–740. 21474434 10.1104/pp.111.176446 PMC3177271

[CIT0056] Jogawat A, Saha S, Bakshi M, Dayaman V, Kumar M, Dua M, Varma A, Oelmüller R, Tuteja N, Johri AK 2013 *Piriformospora indica* rescues growth diminution of rice seedlings during high salt stress. Plant Signaling & Behavior 8, e26891. 10.4161/psb.26891 PMC4091109 24494239

[CIT0057] Johnson JM, Nongbri PL, Sherameti I, Oelmüller R 2011 Calcium signaling and cytosolic calcium measurements in plants. Endocytobiosis and Cell Research 21, 64–76.

[CIT0058] Johnson JM, Thürich J, Petutschnig EK, et al 2018 A poly(A) ribonuclease controls the cellotriose-based interaction between *Piriformospora indica* and its host arabidopsis. Plant Physiology 176, 2496–2514. 29371249 10.1104/pp.17.01423 PMC5841714

[CIT0059] Jumpponen A, Trappe JM 1998 Dark septate endophytes: a review of facultative biotrophic root-colonizing fungi. New Phytologist 140, 295–310. 10.1046/j.1469-8137.1998.00265.x 33862835

[CIT0060] Kadota Y, Shirasu K, Zipfel C 2015 Regulation of the NADPH oxidase RBOHD during plant immunity. Plant & Cell Physiology 56, 1472–1480. 25941234 10.1093/pcp/pcv063

[CIT0061] Khalid M, Rahman SU, Huang D 2019 Molecular mechanism underlying *Piriformospora indica*-mediated plant improvement/protection for sustainable agriculture. Acta Biochimica et Biophysica Sinica 51, 229–242. 30883651 10.1093/abbs/gmz004

[CIT0062] Kiep V, Vadassery J, Lattke J, Maaß JP, Boland W, Peiter E, Mithöfer A 2015 Systemic cytosolic Ca^2+^ elevation is activated upon wounding and herbivory in Arabidopsis. New Phytologist 207, 996–1004. 25996806 10.1111/nph.13493

[CIT0063] Kliebenstein DJ 2016 False idolatry of the mythical growth versus immunity tradeoff in molecular systems plant pathology. Physiological and Molecular Plant Pathology 95, 55–59.

[CIT0064] Kudla J, Becker D, Grill E, Hedrich R, Hippler M, Kummer U, Parniske M, Romeis T, Schumacher K 2018 Advances and current challenges in calcium signaling. New Phytologist 218, 414–431. 29332310 10.1111/nph.14966

[CIT0065] Kumar M, Yadav V, Kumar H, Sharma R, Singh A, Tuteja N, Johri AK 2011 *Piriformospora indica* enhances plant growth by transferring phosphate. Plant Signaling & Behavior 6, 723–725. 21502815 10.4161/psb.6.5.15106 PMC3172848

[CIT0066] Lahrmann U, Strehmel N, Langen G, Frerigmann H, Leson L, Ding Y, Scheel D, Herklotz S, Hilbert M, Zuccaro A 2015 Mutualistic root endophytism is not associated with the reduction of saprotrophic traits and requires a noncompromised plant innate immunity. New Phytologist 207, 841–857. 25919406 10.1111/nph.13411

[CIT0067] Lanza M, Haro R, Conchillo LB, Benito B 2019 The endophyte *Serendipita indica* reduces the sodium content of Arabidopsis plants exposed to salt stress: fungal ENA ATPases are expressed and regulated at high pH and during plant co-cultivation in salinity. Environmental Microbiology 21, 3364–3378. 10.1111/1462-2920.14619 30945789

[CIT0068] Laskowski M, Ten Tusscher KH 2017 Periodic lateral root priming: what makes it tick? The Plant Cell 29, 432–444. 28223442 10.1105/tpc.16.00638 PMC5385950

[CIT0069] Lecourieux D, Mazars C, Pauly N, Ranjeva R, Pugin A 2002 Analysis and effects of cytosolic free calcium increases in response to elicitors in *Nicotiana plumbaginifolia* cells. The Plant Cell 14, 2627–2641. 12368509 10.1105/tpc.005579 PMC151240

[CIT0070] Lee YC, Johnson JM, Chien CT, Sun C, Cai D, Lou B, Oelmüller R, Yeh KW 2011 Growth promotion of Chinese cabbage and *Arabidopsis* by *Piriformospora indica* is not stimulated by mycelium-synthesized auxin. Molecular Plant-Microbe Interactions 24, 421–431. 21375386 10.1094/MPMI-05-10-0110

[CIT0071] Lehmann T, Janowitz T, Sánchez-Parra B, Alonso MP, Trompetter I, Piotrowski M, Pollmann S 2017 Arabidopsis NITRILASE 1 contributes to the regulation of root growth and development through modulation of auxin biosynthesis in seedlings. Frontiers in Plant Science 8, 36. 28174581 10.3389/fpls.2017.00036 PMC5258727

[CIT0072] Leitão N, Dangeville P, Carter R, Charpentier M 2019 Nuclear calcium signatures are associated with root development. Nature Communications 10, 4865. 10.1038/s41467-019-12845-8 PMC6814746 31653864

[CIT0073] Li F, Wang J, Ma C, Zhao Y, Wang Y, Hasi A, Qi Z 2013 Glutamate receptor-like channel3.3 is involved in mediating glutathione-triggered cytosolic calcium transients, transcriptional changes, and innate immunity responses in Arabidopsis. Plant Physiology 162, 1497–1509. 23656893 10.1104/pp.113.217208 PMC3700673

[CIT0074] Li G, Meng X, Wang R, Mao G, Han L, Liu Y, Zhang S 2012 Dual-level regulation of ACC synthase activity by MPK3/MPK6 cascade and its downstream WRKY transcription factor during ethylene induction in Arabidopsis. PLOS Genetics 8, e1002767. 22761583 10.1371/journal.pgen.1002767 PMC3386168

[CIT0075] Li L, Li M, Yu L, et al 2014 The FLS2-associated kinase BIK1 directly phosphorylates the NADPH oxidase RbohD to control plant immunity. Cell Host & Microbe 15, 329–338. 24629339 10.1016/j.chom.2014.02.009

[CIT0076] Liu H, Senthilkumar R, Ma G, Zou Q, Zhu K, Shen X, Tian D, Hua MS, Oelmüller R, Yeh KW 2019 *Piriformospora indica*-induced phytohormone changes and root colonization strategies are highly host-specific. Plant Signaling & Behavior 14, 1632688. 31230564 10.1080/15592324.2019.1632688 PMC6768275

[CIT0077] Lu D, Wu S, Gao X, Zhang Y, Shan L, He P 2010 A receptor-like cytoplasmic kinase, BIK1, associates with a flagellin receptor complex to initiate plant innate immunity. Proceedings of the National Academy of Sciences, USA 107, 496–501. 10.1073/pnas.0909705107 PMC2806711 20018686

[CIT0078] Luck K, Jirschitzka J, Irmisch S, Huber M, Gershenzon J, Köllner TG 2016 CYP79D enzymes contribute to jasmonic acid-induced formation of aldoximes and other nitrogenous volatiles in two *Erythroxylum* species. BMC Plant Biology 16, 215. 27716065 10.1186/s12870-016-0910-5 PMC5050915

[CIT0079] Malla R, Prasad R, Kumari R, Giang PH, Pokharel U, Oelmüller R, Varma A 2004 Phosphorus solubilizing symbiotic fungus: *Piriformospora indica*. Endocytobiosis Cell Research 15, 579–600.

[CIT0080] Manzoor H, Kelloniemi J, Chiltz A, Wendehenne D, Pugin A, Poinssot B, Garcia-Brugger A 2013 Involvement of the glutamate receptor AtGLR3.3 in plant defense signaling and resistance to *Hyaloperonospora arabidopsidis*. The Plant Journal 76, 466–480. 23952652 10.1111/tpj.12311

[CIT0081] McAinsh MR, Pittman JK 2009 Shaping the calcium signature. New Phytologist 181, 275–294. 19121028 10.1111/j.1469-8137.2008.02682.x

[CIT0082] Meents AK, Furch ACU, Almeida-Trapp M, et al 2019 Beneficial and pathogenic Arabidopsis root-interacting fungi differently affect auxin levels and responsive genes during early infection. Frontiers in Microbiology 10, 380. 30915043 10.3389/fmicb.2019.00380 PMC6422953

[CIT0083] Menke FL, van Pelt JA, Pieterse CM, Klessig DF 2004 Silencing of the mitogen-activated protein kinase MPK6 compromises disease resistance in Arabidopsis. The Plant Cell 16, 897–907. 15020743 10.1105/tpc.015552 PMC412864

[CIT0084] Molina-Montenegro MA, Oses R, Torres-Diaz C, Atala C, Zurita-Silva A, Ruiz-Lara S 2016 Root-endophytes improve the ecophysiological performance and production of an agricultural species under drought condition. AoB Plants 8, plw062. 27613875 10.1093/aobpla/plw062 PMC5091693

[CIT0085] Mravec J, Skůpa P, Bailly A, et al 2009 Subcellular homeostasis of phytohormone auxin is mediated by the ER-localized PIN5 transporter. Nature 459, 1136–1140. 19506555 10.1038/nature08066

[CIT0086] Murray DR 1997 Carbon dioxide and plant responses. Taunton, Research Studies Press.

[CIT0087] Nizam S, Qiang X, Wawra S, Nostadt R, Getzke F, Schwanke F, Dreyer I, Langen G, Zuccaro A 2019 *Serendipita indica* E5′NT modulates extracellular nucleotide levels in the plant apoplast and affects fungal colonization. EMBO Reports 20, e47430. 30642845 10.15252/embr.201847430 PMC6362346

[CIT0088] Nongbri PL, Johnson JM, Sherameti I, Glawischnig E, Halkier BA, Oelmüller R 2012 Indole-3-acetaldoxime-derived compounds restrict root colonization in the beneficial interaction between *Arabidopsis* roots and the endophyte *Piriformospora indica*. Molecular Plant-Microbe Interactions 25, 1186–1197. 22852809 10.1094/MPMI-03-12-0071-R

[CIT0089] Nühse TS, Peck SC, Hirt H, Boller T 2000 Microbial elicitors induce activation and dual phosphorylation of the *Arabidopsis thaliana* MAPK 6. Journal of Biological Chemistry 275, 7521–7526. 10713056 10.1074/jbc.275.11.7521

[CIT0090] Oelmüller R 2018 Sensing environmental and developmental signals via cellooligomers. Journal of Plant Physiology 229, 1–6. 30005268 10.1016/j.jplph.2018.06.010

[CIT0091] Oelmüller R, Sherameti I, Tripathi S, Varma A 2009 *Piriformospora indica*, a cultivable root endophyte with multiple biotechnological applications. Symbiosis 49, 1–17.

[CIT0092] Overvoorde P, Fukaki H, Beeckman T 2010 Auxin control of root development. Cold Spring Harbor Perspectives in Biology 2, a001537. 20516130 10.1101/cshperspect.a001537 PMC2869515

[CIT0093] Peiter E, Maathuis FJ, Mills LN, Knight H, Pelloux J, Hetherington AM, Sanders D 2005 The vacuolar Ca^2+^-activated channel TPC1 regulates germination and stomatal movement. Nature 434, 404–408. 15772667 10.1038/nature03381

[CIT0094] Peškan-Berghöfer T, Shahollari B, Giong PH, Hehl S, Markert C, Blanke V, Kost G, Varma A, Oelmüller R 2004 Association of *Piriformospora indica* with *Arabidopsis thaliana* roots represents a novel system to study beneficial plant–microbe interactions and involves early plant protein modifications in the endoplasmic reticulum and at the plasma membrane. Physiologia Plantarum 122, 465–477.

[CIT0095] Petit JR, Jouzel J, Raynaud D, et al 1999 Climate and atmospheric history of the past 420,000 years from the Vostok ice core, Antarctica. Nature 399, 429–436.

[CIT0096] Pollmann S, Müller A, Weiler EW 2006 Many roads lead to “auxin”: of nitrilases, synthases, and amidases. Plant Biology 8, 326–333. 16807824 10.1055/s-2006-924075

[CIT0097] Qiang X, Weiss M, Kogel KH, Schäfer P 2012a *Piriformospora indica*—a mutualistic basidiomycete with an exceptionally large plant host range. Molecular Plant Pathology 13, 508–518. 22111580 10.1111/j.1364-3703.2011.00764.x PMC6638644

[CIT0098] Qiang X, Zechmann B, Reitz MU, Kogel KH, Schäfer P 2012b The mutualistic fungus *Piriformospora indica* colonizes *Arabidopsis* roots by inducing an endoplasmic reticulum stress-triggered caspase-dependent cell death. The Plant Cell 24, 794–809. 22337916 10.1105/tpc.111.093260 PMC3315247

[CIT0099] Ramos P, Rivas N, Pollmann S, Casati P, Molina-Montenegro MA 2018 Hormonal and physiological changes driven by fungal endophytes increase Antarctic plant performance under UV-B radiation. Fungal Ecology 34, 76–82.

[CIT0100] Ren D, Liu Y, Yang KY, Han L, Mao G, Glazebrook J, Zhang S 2008 A fungal-responsive MAPK cascade regulates phytoalexin biosynthesis in *Arabidopsis*. Proceedings of the National Academy of Sciences, USA 105, 5638–5643. 10.1073/pnas.0711301105 PMC2291085 18378893

[CIT0101] Rennenberg H, Loreto F, Polle A, Brilli F, Fares S, Beniwal RS, Gessler A 2006 Physiological responses of forest trees to heat and drought. Plant Biology 8, 556–571. 16773557 10.1055/s-2006-924084

[CIT0102] Rodriguez RJ, Henson J, Van Volkenburgh E, Hoy M, Wright L, Beckwith F, Kim YO, Redman RS 2008 Stress tolerance in plants via habitat-adapted symbiosis. The ISME Journal 2, 404–416. 18256707 10.1038/ismej.2007.106

[CIT0103] Rodriguez RJ, Redman RS, Henson JM 2004 The role of fungal symbioses in the adaptation of plants to high stress environments. Mitigation and Adaptation Strategies for Global Change 9, 261–272.

[CIT0104] Roelfsema MR, Hedrich R 2005 In the light of stomatal opening: new insights into ‘the Watergate’. New Phytologist 167, 665–691. 16101906 10.1111/j.1469-8137.2005.01460.x

[CIT0105] Ruiz-Lozano JM, Porcel R, Azcón C, Aroca R 2012 Regulation by arbuscular mycorrhizae of the integrated physiological response to salinity in plants: new challenges in physiological and molecular studies. Journal of Experimental Botany 63, 4033–4044. 22553287 10.1093/jxb/ers126

[CIT0106] Saddique MAB, Ali Z, Khan AS, Rana IA, Shamsi IH 2018 Inoculation with the endophyte *Piriformospora indica* significantly affects mechanisms involved in osmotic stress in rice. Rice 11, 34. 29799607 10.1186/s12284-018-0226-1 PMC5968016

[CIT0107] Sade N, Gebretsadik M, Seligmann R, Schwartz A, Wallach R, Moshelion M 2010 The role of tobacco Aquaporin1 in improving water use efficiency, hydraulic conductivity, and yield production under salt stress. Plant Physiology 152, 245–254. 19939947 10.1104/pp.109.145854 PMC2799360

[CIT0108] Schäfer P, Pfiffi S, Voll LM, et al 2009 Manipulation of plant innate immunity and gibberellin as factor of compatibility in the mutualistic association of barley roots with *Piriformospora indica*. The Plant Journal 59, 461–474. 19392709 10.1111/j.1365-313X.2009.03887.x

[CIT0109] Schroeder JI, Kwak JM, Allen GJ 2001 Guard cell abscisic acid signalling and engineering drought hardiness in plants. Nature 410, 327–330. 11268200 10.1038/35066500

[CIT0110] Schuman MC, Baldwin IT 2016 The layers of plant responses to insect herbivores. Annual Review of Entomology 61, 373–394. 10.1146/annurev-ento-010715-023851 26651543

[CIT0111] Shahollari B, Varma A, Oelmüller R 2005 Expression of a receptor kinase in *Arabidopsis* roots is stimulated by the basidiomycete *Piriformospora indica* and the protein accumulates in Triton X-100 insoluble plasma membrane microdomains. Journal of Plant Physiology 162, 945–958. 16146321 10.1016/j.jplph.2004.08.012

[CIT0112] Sharma P, Kharkwal AC, Abdin MZ, Varma A 2014 *Piriformospora indica* improves micropropagation, growth and phytochemical content of *Aloe vera* L. plants. Symbiosis 64, 11–23.

[CIT0113] Sherameti I, Shahollari B, Venus Y, Altschmied L, Varma A, Oelmüller R 2005 The endophytic fungus *Piriformospora indica* stimulates the expression of nitrate reductase and the starch-degrading enzyme glucan-water dikinase in tobacco and *Arabidopsis* roots through a homeodomain transcription factor that binds to a conserved motif in their promoters. Journal of Biological Chemistry 280, 26241–26247. 15710607 10.1074/jbc.M500447200

[CIT0114] Sherameti I, Tripathi S, Varma A, Oelmüller R 2008 The root-colonizing endophyte *Pirifomospora indica* confers drought tolerance in *Arabidopsis* by stimulating the expression of drought stress–related genes in leaves. Molecular Plant-Microbe Interactions 21, 799–807. 18624643 10.1094/MPMI-21-6-0799

[CIT0115] Shi H, Shen Q, Qi Y, Yan H, Nie H, Chen Y, Zhao T, Katagiri F, Tang D 2013 BR-SIGNALING KINASE1 physically associates with FLAGELLIN SENSING2 and regulates plant innate immunity in *Arabidopsis*. The Plant Cell 25, 1143–1157. 23532072 10.1105/tpc.112.107904 PMC3634682

[CIT0116] Shinozaki K, Yamaguchi-Shinozaki K 2007 Gene networks involved in drought stress response and tolerance. Journal of Experimental Botany 58, 221–227. 17075077 10.1093/jxb/erl164

[CIT0117] Souza CA, Li S, Lin AZ, Boutrot F, Grossmann G, Zipfel C, Somerville SC 2017 Cellulose-derived oligomers act as damage-associated molecular patterns and trigger defense-like responses. Plant Physiology 173, 2383–2398. 28242654 10.1104/pp.16.01680 PMC5373054

[CIT0118] Spaepen S, Vanderleyden J, Remans R 2007 Indole-3-acetic acid in microbial and microorganism-plant signaling. FEMS Microbiology Reviews 31, 425–448. 17509086 10.1111/j.1574-6976.2007.00072.x

[CIT0119] Staswick PE, Serban B, Rowe M, Tiryaki I, Maldonado MT, Maldonado MC, Suza W 2005 Characterization of an Arabidopsis enzyme family that conjugates amino acids to indole-3-acetic acid. The Plant Cell 17, 616–627. 15659623 10.1105/tpc.104.026690 PMC548830

[CIT0120] Strehmel N, Monchgesang S, Herklotz S, Kruger S, Ziegler J, Scheel D 2016 *Piriformospora indica* stimulates root metabolism of *Arabidopsis thaliana*. International Journal of Molecular Sciences 17, 1091. 10.3390/ijms17071091 PMC4964467 27399695

[CIT0121] Su ZZ, Wang T, Shrivastava N, Chen YY, Liu X, Sun C, Yin Y, Gao QK, Lou BG 2017 *Piriformospora indica* promotes growth, seed yield and quality of *Brassica napus* L. Microbiological Research 199, 29–39. 28454707 10.1016/j.micres.2017.02.006

[CIT0122] Sun C, Shao Y, Vahabi K, et al 2014 The beneficial fungus *Piriformospora indica* protects Arabidopsis from *Verticillium dahliae* infection by downregulation plant defense responses. BMC Plant Biology 14, 268. 25297988 10.1186/s12870-014-0268-5 PMC4198706

[CIT0123] Takahashi F, Yoshida R, Ichimura K, Mizoguchi T, Seo S, Yonezawa M, Maruyama K, Yamaguchi-Shinozaki K, Shinozaki K 2007 The mitogen-activated protein kinase cascade MKK3–MPK6 is an important part of the jasmonate signal transduction pathway in *Arabidopsis*. The Plant Cell 19, 805–818. 17369371 10.1105/tpc.106.046581 PMC1867372

[CIT0124] Thürich J, Meichsner D, Furch ACU, Pfalz J, Krüger T, Kniemeyer O, Brakhage A, Oelmüller R 2018 *Arabidopsis thaliana* responds to colonisation of *Piriformospora indica* by secretion of symbiosis-specific proteins. PLoS One 13, e0209658. 30589877 10.1371/journal.pone.0209658 PMC6307754

[CIT0125] Timilsena YP, Adhikari R, Casey P, Muster T, Gill H, Adhikari B 2015 Enhanced efficiency fertilisers: a review of formulation and nutrient release patterns. Journal of the Science of Food and Agriculture 95, 1131–1142. 25043832 10.1002/jsfa.6812

[CIT0126] Upadhyaya CP, Gururani MA, Prasad R, Verma A 2013 A cell wall extract from *Piriformospora indica* promotes tuberization in potato (*Solanum tuberosum* L.) via enhanced expression of Ca^+2^ signaling pathway and lipoxygenase gene. Applied Biochemistry and Biotechnology 170, 743–755. 23609909 10.1007/s12010-013-0231-1

[CIT0127] Vadassery J, Ranf S, Drzewiecki C, Mithöfer A, Mazars C, Scheel D, Lee J, Oelmüller R 2009 A cell wall extract from the endophytic fungus *Piriformospora indica* promotes growth of Arabidopsis seedlings and induces intracellular calcium elevation in roots. The Plant Journal 59, 193–206. 19392691 10.1111/j.1365-313X.2009.03867.x

[CIT0128] Vadassery J, Ritter C, Venus Y, Camehl I, Varma A, Shahollari B, Novák O, Strnad M, Ludwig-Müller J, Oelmüller R 2008 The role of auxins and cytokinins in the mutualistic interaction between *Arabidopsis* and *Piriformospora indica*. Molecular Plant-Microbe Interactions 21, 1371–1383. 18785832 10.1094/MPMI-21-10-1371

[CIT0129] Vahabi K, Sherameti I, Bakshi M, Mrozinska A, Ludwig A, Reichelt M, Oelmüller R 2015 The interaction of Arabidopsis with *Piriformospora indica* shifts from initial transient stress induced by fungus-released chemical mediators to a mutualistic interaction after physical contact of the two symbionts. BMC Plant Biology 15, 58. 25849363 10.1186/s12870-015-0419-3 PMC4384353

[CIT0130] van’t Padje A, Whiteside MD, Kiers ET 2016 Signals and cues in the evolution of plant-microbe communication. Current Opinion in Plant Biology 32, 47–52. 27348594 10.1016/j.pbi.2016.06.006

[CIT0131] Varma A, Savita Verma, Sudha, Sahay N, Butehorn B, Franken P 1999 *Piriformospora indica*, a cultivable plant-growth-promoting root endophyte. Applied and Environmental Microbiology 65, 2741–2744. 10347070 10.1128/aem.65.6.2741-2744.1999 PMC91405

[CIT0132] Verma S, Varma A, Rexer K-H, Hassel A, Kost G, Sarbhoy A, Bisen P, Bütehorn B, Franken P 1998 *Piriformospora indica*, gen. et sp. nov., a new root-colonizing fungus. Mycologia 90, 896–903.

[CIT0133] Waller F, Achatz B, Baltruschat H, et al 2005 The endophytic fungus *Piriformospora indica* reprograms barley to salt-stress tolerance, disease resistance, and higher yield. Proceedings of the National Academy of Sciences, USA 102, 13386–13391. 10.1073/pnas.0504423102 PMC1224632 16174735

[CIT0134] Wang W, Shi J, Xie Q, Jiang Y, Yu N, Wang E 2017 Nutrient exchange and regulation in arbuscular mycorrhizal symbiosis. Molecular Plant 10, 1147–1158. 28782719 10.1016/j.molp.2017.07.012

[CIT0135] Weiss M, Selosse MA, Rexer KH, Urban A, Oberwinkler F 2004 *Sebacinales*: a hitherto overlooked cosm of heterobasidiomycetes with a broad mycorrhizal potential. Mycological Research 108, 1003–1010. 15506013 10.1017/s0953756204000772

[CIT0136] Weiß M, Sýkorová Z, Garnica S, Riess K, Martos F, Krause C, Oberwinkler F, Bauer R, Redecker D 2011 Sebacinales everywhere: previously overlooked ubiquitous fungal endophytes. PLoS One 6, e16793. 21347229 10.1371/journal.pone.0016793 PMC3039649

[CIT0137] Weiß M, Waller F, Zuccaro A, Selosse MA 2016 Sebacinales - one thousand and one interactions with land plants. New Phytologist 211, 20–40. 27193559 10.1111/nph.13977

[CIT0138] Yadav V, Kumar M, Deep DK, Kumar H, Sharma R, Tripathi T, Tuteja N, Saxena AK, Johri AK 2010 A phosphate transporter from the root endophytic fungus *Piriformospora indica* plays a role in phosphate transport to the host plant. Journal of Biological Chemistry 285, 26532–26544. 20479005 10.1074/jbc.M110.111021 PMC2924090

[CIT0139] Zhang J, Li W, Xiang T, et al 2010 Receptor-like cytoplasmic kinases integrate signaling from multiple plant immune receptors and are targeted by a *Pseudomonas syringae* effector. Cell Host & Microbe 7, 290–301. 20413097 10.1016/j.chom.2010.03.007

[CIT0140] Zhang W, Wang J, Xu L, Wang A, Huang L, Du H, Qiu L, Oelmüller R 2018 Drought stress responses in maize are diminished by *Piriformospora indica*. Plant Signaling & Behavior 13, e1414121. 29219729 10.1080/15592324.2017.1414121 PMC5790412

[CIT0141] Zipfel C, Oldroyd GE 2017 Plant signalling in symbiosis and immunity. Nature 543, 328–336. 28300100 10.1038/nature22009

[CIT0142] Zuccaro A, Basiewicz M, Zurawska M, Biedenkopf D, Kogel KH 2009 Karyotype analysis, genome organization, and stable genetic transformation of the root colonizing fungus *Piriformospora indica*. Fungal Genetics and Biology 46, 543–550. 19351564 10.1016/j.fgb.2009.03.009

[CIT0143] Zuccaro A, Lahrmann U, Güldener U, et al 2011 Endophytic life strategies decoded by genome and transcriptome analyses of the mutualistic root symbiont *Piriformospora indica*. PLOS Pathogens 7, e1002290. 22022265 10.1371/journal.ppat.1002290 PMC3192844

[CIT0144] Züst T, Rasmann S, Agrawal AA 2015 Growth–defense tradeoffs for two major anti-herbivore traits of the common milkweed *Asclepias syriaca*. Oikos 124, 1404–1415.

